# Management of Ledge Formation and Perforations in Endodontics: A Survey of Knowledge, Attitudes, Practices, and Perceptions Among General Dental Practitioners and Endodontists

**DOI:** 10.4317/jced.64141

**Published:** 2026-05-29

**Authors:** Aruna Kumari Veronica, Kumar Dhanush, Venkatachalam Prakash, Anand Susila

**Affiliations:** 1 MDS, PhD Research scholar. Department of Conservative Dentistry and Endodontics, Bharath Institute of Higher Education and Research, Selaiyur, Chennai. 600100. Professor, Madha Dental College and Hospital, Chennai, Tamil Nadu, Chennai, India 600069; 2 BDS, PG student. Department of Conservative Dentistry &amp; Endodontics, Madha Dental College &amp; Hospital, Chennai, Tamil nadu, India. 600069; 3 MDS, Professor. Department of Conservative Dentistry and Endodontics, Shree Balaji Dental College and Hospital-Chennai. 600100; 4 MDS, PhD, Professor and Head. Department of Conservative Dentistry &amp; Endodontics, Madha Dental College &amp; Hospital, Chennai, Tamil nadu, India. 600069

## Abstract

**Background:**

This study compared knowledge, attitudes, and perceptions of endodontists and general practitioners regarding ledge formation and root perforation.

**Materials and Methods:**

A 15-item questionnaire assessed knowledge, attitudes, perceptions, and practices on ledge formation and root perforation. It was pilot-tested on 20 endodontists, revised for clarity and validity, and finalized for data collection.

**Results:**

A total of 202 responses (65%) were analyzed, including 51% academicians and 49% private practitioners. Most participants (82%) identified stiff instruments in curved canals as the primary cause of ledge formation, while 80% recognized sudden loss of working length as a key sign; 59% identified causes of apical perforation. Mineral trioxide aggregate was preferred by 98% for perforation repair, and CBCT by 72% for diagnosis. Additionally, 95% agreed that Ni-Ti instruments help prevent ledge formation, 84% practiced pre-curving of files, and 72% prioritized sealing, while 98% supported referral of perforation cases. Despite strong knowledge, clinical gaps were evident, with only 46% consistently using apex locators and 60% adhering to recommended protocols. Significant associations by experience involved causes, Ni-Ti use, apex locator use, management goals, and tip design. By professional status, differences included causes, Ni-Ti use, pre-curving, apex locator use, techniques, and rake angle. By practice type, causes, pre-curving, and rake angle varied. The Chi-square (²) test was used, with p &lt; 0.05 considered statistically significant.

**Conclusions:**

While practitioners demonstrate strong knowledge and positive attitudes, gaps in clinical implementation persist, particularly in the use of apex locators, adherence to protocols, and consistency in understanding causative factors.

## Introduction

Endodontic mishaps such as ledge formation and root perforation are predominantly iatrogenic and may compromise treatment outcomes. Ledge formation is defined as an artificial irregularity or deviation created on the canal wall that alters the original canal curvature, whereas root perforation refers to a pathological or iatrogenic communication between the root canal system and the external root surface (1 , 2). The etiology of these mishaps is multifactorial and may occur during any stage of root canal treatment, including access preparation, cleaning and shaping, and obturation. These endodontic mishaps are associated with extensive caries, root resorption, extracoronal restorations, intracanal posts, misidentification of root canal anatomy, curved canals, and procedural errors during root canal preparation (1 - 4). Among procedural errors, ledge formation is one of the most frequently encountered mishaps, with a reported incidence of approximately 47% (5). Stainless steel instruments, due to their stiffness and limited flexibility, tend to straighten within curved canals, thereby increasing the risk of ledge formation and canal transportation (6). Conventional NiTi instruments exhibit superelasticity and improved canal-centering ability compared with stainless steel files (7). However, newer thermomechanically treated NiTi systems such as HyFlex CM, HyFlex EDM, BT-Race, Vortex Blue, ProTaper Gold, XP-endo Shaper, and One Curve offer greater flexibility and fatigue resistance, making them more suitable for bypassing ledges and negotiating complex canal anatomy (8). Management of ledges commonly involves bypassing the obstruction using pre-curved stainless steel K-files directed toward the inner canal curvature (9). The reported prevalence of root perforation ranges from 0.6% to 17.6%, with tooth morphology, canal anatomy, and operator experience identified as major contributing factors (10). Although periapical radiographs remain routinely used for diagnosis, advanced modalities such as electronic apex locators (EALs), dental operating microscopes, and cone-beam computed tomography (CBCT) provide improved accuracy in detecting perforations and procedural errors (11 - 13). However, EAL accuracy may be affected by irrigants such as sodium hypochlorite (14). The prognosis of perforations depends largely on the size, location, timing of repair, and the ability to achieve immediate sealing with a biocompatible material (2). Historically, materials such as amalgam, glass ionomer cement, zinc oxide-eugenol, gutta-percha, composite resin, Super EBA, and Cavit have been used for perforation repair. More recently, bioactive materials including mineral trioxide aggregate (MTA), Biodentine, BioAggregate, calcium-enriched mixture (CEM), EndoSequence Root Repair Material, iRoot BP, calcium hydroxide, calcium phosphate-based materials, collagen, and resin cements have gained prominence because of their superior sealing ability and biocompatibility. Among these, MTA is widely regarded as the gold standard for perforation repair (2). Knowledge, attitudes, and perceptions of dental professionals regarding ledge formation and root perforation are important, as awareness of etiological factors and management strategies may vary with clinical experience. This survey was conducted to assess and compare the knowledge, attitudes, and perceptions of endodontists and general dental practitioners regarding ledge formation and root perforation.

## Materials and Methods

- Ethical Considerations The study protocol was approved by the Institutional Ethical Board (Ref. No: IRB/MDCH/2026/26-15). Participation was voluntary, and informed consent was obtained electronically before participation. Responses were collected anonymously and maintained confidentially throughout the study. - Study Design and Setting A nationwide cross-sectional survey was conducted among general dental practitioners and endodontists involved in academic and/or clinical practice to assess their knowledge, perceptions, attitudes, and clinical practices related to ledge formation and perforation. The survey was administered electronically using Google Forms and distributed through professional dental associations across India. - Sampling strategy and participant recruitment A non-probability convenience sampling method with snowball dissemination was employed for participant recruitment. The survey link was circulated through professional networks, email groups, and social media platforms affiliated with the Indian Dental Association, Indian Association of Conservative Dentistry and Endodontics, and Indian Endodontic Society. Participants were encouraged to share the survey with eligible colleagues to improve national outreach and response representation. Several measures were implemented to minimize potential bias. Participation was voluntary and anonymous to reduce social desirability bias. Duplicate responses were excluded through timestamp and response-pattern verification. Mandatory response settings were enabled to reduce missing data, and participants were restricted to a single response to minimize response duplication and selection bias. - Inclusion and Exclusion Criteria: Inclusion criteria included general dental practitioners and endodontists practicing in India, involved in academic practice, clinical practice, or both, and willing to provide informed consent electronically. Exclusion criteria included undergraduate students, interns, incomplete or duplicate responses, dental professionals practicing outside India, specialists from other dental disciplines, and participants unwilling to provide informed consent. - Questionnaire Validation and Reliability The questionnaire was developed following an extensive literature review on ledge formation and perforation during endodontic treatment. It consisted of 15 multiple-choice questions assessing knowledge (5 questions), clinical practice (4 questions), perception (3 questions), and attitude (3 questions). Face and content validity were evaluated by a panel of experienced endodontists and academicians for relevance, clarity, and comprehensiveness. A pilot study was conducted among 20 endodontists who were excluded from the final sample. Based on their feedback, necessary modifications were made to improve clarity and eliminate ambiguity. Internal consistency reliability was assessed using Cronbach's alpha coefficient before final administration of the questionnaire. - Data Collection Data were collected using a structured self-administered questionnaire hosted on Google Forms. Participants were allowed to respond only once, and no personally identifiable information was collected. Mandatory response settings were enabled to minimize missing data. Knowledge-based questions assessed diagnosis, etiological factors, signs and symptoms, and material selection for perforation repair. Practice-based questions evaluated the use of electronic apex locators, canal preparation techniques, pre-curving of hand instruments, and management objectives for apical perforations. Perception-based questions explored opinions regarding instruments and instrument designs associated with ledge formation, while attitude-based questions assessed perceptions regarding causes, prevention, and management of perforation cases. - Variables and Subgroup Analysis Demographic and professional variables collected included type of practice (academic or private), professional status (general dental practitioner or endodontist), and years of clinical experience. Participants were categorized into four groups according to clinical experience: &lt;1 year, 1-5 years, 6-10 years, and &gt;10 years. These variables were analyzed to assess differences in knowledge, perception, attitude, and clinical practice patterns among subgroups. - Statistical Analysis Data were entered into Microsoft Excel for organization and cleaning and analyzed using IBM SPSS Statistics software. Descriptive statistics were used to summarize the data. Associations between categorical variables were assessed using the Chi-square (²) test. A p-value &lt;0.05 was considered statistically significant.

## Results

**Figure 1:**
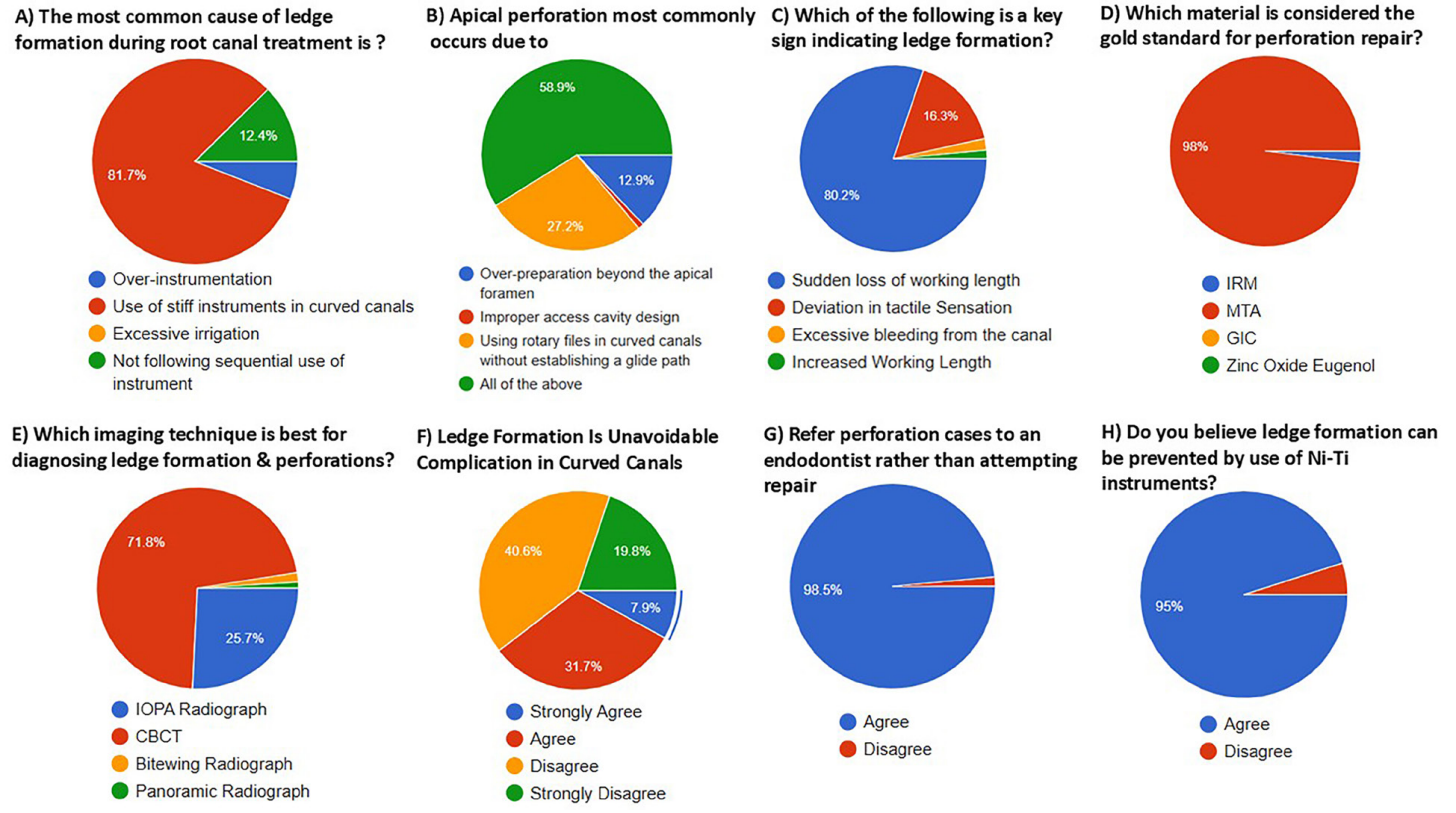
Distribution of responses for knowledge and attitude domains.

A total of 202 responses were obtained (response rate: 65%) from members of the Indian Dental Association, Indian Association of Conservative Dentistry and Endodontics, and Indian Endodontic Society. Among the participants, 105 were general practitioners and 97 were endodontists/postgraduates. Of the respondents, 51% worked in academic institutions and 49% in private practice. Clinical experience distribution was 13.9% (&lt;1 year), 54.5% (1-5 years), 20.3% (6-10 years), and 11.4% (&gt;10 years). - Knowledge Most respondents (81.7%) identified use of stiff instruments in curved canals as the most common cause of ledge formation (Fig. 1A).

1

**Figure 2:**
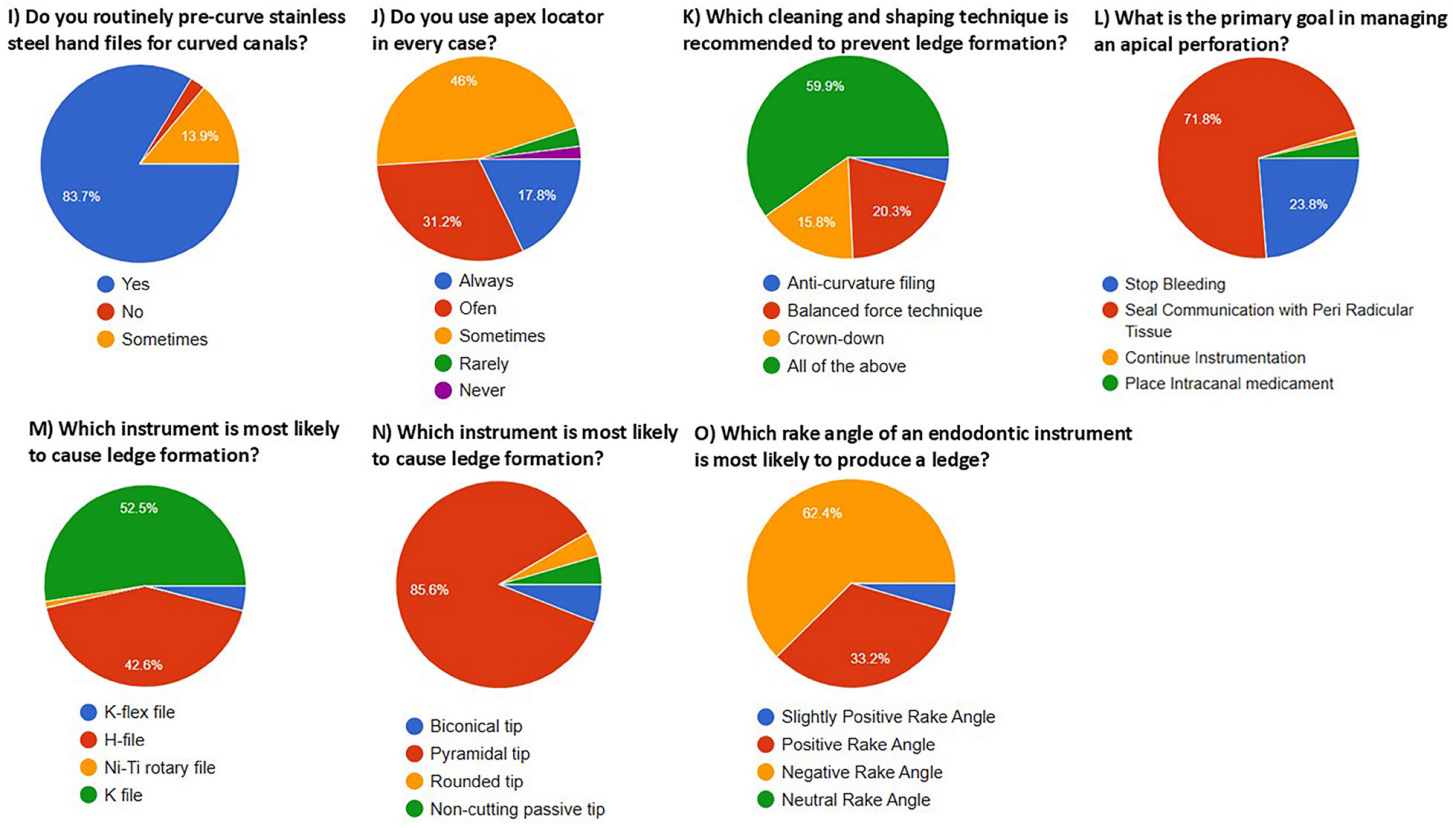
Distribution of response for practice and perception domain.

Fifty-nine percent correctly identified all listed factors contributing to apical perforation, including rotary instrumentation in curved canals without glide path establishment, improper access preparation, and overpreparation (Fig. 1B). Sudden loss of working length was recognized as the key sign of ledge formation by 80% (Fig. 1C). Mineral trioxide aggregate was identified as the gold standard for perforation repair by 98% (Fig. 1D), and cone-beam computed tomography was preferred for diagnosis by 72%(Fig. 1E). - Attitude Ledge formation is unavoidable in curved canal was disagreed by 41% (Fig. 1F), Most respondents agreed that perforation cases should be referred to an endodontist (98%) (Fig. 1G), and that nickel-titanium instruments help prevent ledge formation (95%) (Fig. 1H). - Practice Pre-curving of stainless steel hand files was routinely practiced by 84% (Fig. 2I), while 46% reported occasional use of an electronic apex locator (Fig. 2J).

2

Anticurvature filing, balanced force technique, and crown-down preparation were followed by 60% (Fig. 2K). Sealing communication with periradicular tissues was considered the primary objective in managing apical perforations by 72% (Fig. 2L). Perception K-files were perceived as the instrument most likely to cause ledge formation (52%) (Fig. 2M). A pyramidal tip design (86%) (Fig 2N) and negative rake angle (62%) (Fig. 2O) were most frequently identified as instrument features associated with ledge formation. Based on years of experience: significant associations were observed for the cause of ledge formation (p = 0.001), belief in Ni-Ti instruments preventing ledge formation (p = 0.001), apex locator use (p = 0.049), primary goal in apical perforation management (p = 0.02), and tip design associated with ledge formation (p = 0.02)(Table 1).

Table 1

Based on professional status: significant differences were noted for the cause of ledge formation (p = 0.030), causes of apical perforation (p = 0.001), belief in Ni-Ti instruments (p = 0.036), pre-curving of stainless steel files (p = 0.024), apex locator use (p = 0.001), recommended cleaning and shaping technique (p = 0.001), and rake angle associated with ledge formation (p = 0.006)(Table 2).

Table 2

Based on type of practice: significant associations were observed for the cause of ledge formation (p = 0.044), pre-curving of stainless steel hand files (p = 0.042), and rake angle associated with ledge formation (p = 0.008)(Table 3).

Table 3

## Discussion

This study provides insight into the knowledge, attitudes, and clinical practices of dental practitioners and endodontists regarding endodontic mishaps, particularly ledge formation and apical perforation. Greater knowledge among endodontists and postgraduate practitioners highlights the influence of specialized training and clinical experience. The findings further emphasize that these procedural errors are multifactorial and associated with inadequate understanding of root canal morphology, improper access preparation, inaccurate working length determination, and inappropriate instrumentation techniques, particularly in curved canals. In the present survey, 81.7% of participants identified the use of stiff instruments in curved canals as the primary cause of ledge formation. Clinically, NiTi rotary instruments are more efficient than hand instrumentation because stress-induced transformation from austenite to martensite allows greater flexibility and stress accommodation (1 , 8). Other recognized etiologic factors included skipping instrument sequences and over-instrumentation of the apical third (1). Knowledge levels varied with clinical training, professional status, and experience. Endodontic residents and specialists demonstrated greater awareness of appropriate cleaning and shaping protocols, particularly the importance of maintaining instrument sequence. Clinicians with 6-10 years of experience more frequently selected correct responses. About 60% of participants correctly identified factors associated with apical perforation. Establishing a glide path is essential for minimizing endodontic mishaps by creating a smooth, reproducible pathway from the canal orifice to the apical terminus. Glide path preparation can be achieved using hand K-files, hand K-files in a reciprocating handpiece, or rotary NiTi files. Preventive strategies include coronal preflaring, glide path establishment, maintenance of apical patency, and restricting instrumentation within the working length (15). Inaccurate working length determination and improper access preparation increase the risk of over-instrumentation, ledge formation, and perforation (16). Endodontists and postgraduate practitioners demonstrated greater awareness than general practitioners, particularly regarding incorrect working length as a cause of apical perforation. More than three-fourths of respondents correctly identified sudden loss of working length as a key indicator of ledge formation. In curved canals, underextended obturation is commonly associated with ledge formation (17). Nearly all participants selected mineral trioxide aggregate (MTA) for perforation repair because of its bioactivity, sealing ability, and non-resorbable properties, making it the gold standard material (18). About 72% recognized cone-beam computed tomography (CBCT) as a reliable tool for detecting procedural errors through accurate three-dimensional assessment of canal anatomy and perforation sites (19). A previous Brazilian survey reported CBCT use by 53.9% of endodontists for perforation management (20). No statistically significant differences were found in knowledge-based responses across years of experience, professional status, or practice type. Approximately 41% of participants disagreed with the statement that ledge formation is unavoidable in curved canals. According to the AAE Endodontic Case Difficulty Assessment Guidelines, cases with minimal curvature (&lt;10°) are classified as minimal difficulty (average risk), those with moderate curvature (10°-30°) as moderate difficulty (high risk), and those with severe curvature (&gt;30°) or S-shaped canals as high difficulty (very high risk) (7). Nearly all participants agreed that perforation cases warrant referral to a specialist, reflecting the technical complexity and expertise required for their management. No significant differences were observed across years of clinical experience, professional status, or practice type for these attitude-based responses. Most respondents agreed that NiTi instruments helps prevent ledge formation. They preserve the original canal curvature, reducing procedural errors such as ledging, canal transportation, and perforation (1 , 16 , 17). Recent advances, including controlled memory alloys, EDM technology, electropolishing, martensitic TiO2 surface treatment, and MaxWire, T-wire, and C-wire systems, further improve flexibility and fatigue resistance, thereby minimizing mishaps in curved canals and aiding ledge bypassing (8). This perception showed significant associations with years of clinical experience and professional status, but not with type of practice. The differences observed between specialists and general practitioners may reflect specialists' broader understanding of additional contributing factors, including access preparation, working length determination, instrument design, and shaping techniques (1 , 17 , 21). Approximately 84% of respondents reported pre-curving stainless steel instruments when preparing curved canals, indicating awareness of strategies to minimize procedural errors. In such canals, pre-curving stiff instruments helps maintain canal anatomy and achieve full working length. Small K-files (sizes 10 or 15) are typically pre-curved either with a gentle continuous curvature along the file length or with a sharp 45° bend near the apical tip (22 , 15). This practice showed a statistically significant association with professional status and type of practice, whereas years of experience had no significant effect. A minority of specialists reported not pre-curving hand files, likely reflecting the use of rotary glide path systems, which improve efficiency, reduce operator fatigue, and maintain canal morphology with minimal procedural errors and apical debris extrusion (15). Nearly half of participants reported only occasional use of electronic apex locators (EALs). Although electronic apex locators (EALs) are widely recognized for accurately identifying the apical constriction and are often considered more reliable than radiographic methods for working length determination (12), other studies have reported that both methods are equally effective (24 , 25). In an umbrella review, Pisano reported that among five systematic reviews comparing EALs and radiographic methods, two found no significant difference, one favored EALs, one favored radiographic methods, and one found CBCT to be comparable to EALs (24). EALs were used in 95% of undergraduate endodontic teaching programs in Spain (26). In the present study, EAL use was significantly associated with years of experience and professional status, but not with type of practice. These findings highlight the role of clinical experience and training in the adoption of endodontic technologies. Approximately 60% of participants reported using anti-curvature filing, crown-down preparation, and the balanced force technique. Evidence suggests these approaches reduce procedural errors. Anti-curvature filing directs instrumentation away from the thinner inner canal wall, minimizing the risk of ledge formation and perforation in severely curved canals (27). The balanced force technique enhances control and maintains original canal curvature, thereby reducing mishaps such as ledging and perforation (21 , 22). Crown-down preparation facilitates early coronal flaring, improving irrigation, reducing debris accumulation, and lowering ledging incidence (27 , 28). When stratified by professional status, over 70% of endodontists reported appropriate preparation techniques compared with approximately half of general practitioners, underscoring the role of training in adopting evidence-based practices. Regarding the primary treatment objective of apical perforations, 72% of participants correctly identified sealing the communication as the main goal. Previous studies have demonstrated that MTA is superior to amalgam, Epiphany, and GIC for perforation repair owing to its superior sealing ability and resistance to microbial leakage, which are critical for healing in endodontic procedures (29). Recent studies have further reported that light-cure MTA exhibits improved sealing ability relative to conventional MTA and Biodentine (30). Greater awareness of this concept was observed among clinicians with (&lt; 6 years) of experience and trained endodontists. Most participants identified K-files as the instruments most commonly associated with ledge formation, followed by H-files, K-Flex files, and NiTi rotary instruments. This finding is consistent with published literature reporting a higher incidence of ledge formation with hand K-files compared with NiTi rotary systems (1). NiTi rotary files have lower modulus of elasticity and exerts fewer lateral forces on dentinal walls. The design of H-files predisposes them to procedural mishaps, whereas K-Flex files were developed to reduce complications such as ledge formation and perforation. Responses to this question were not significantly influenced by clinical experience, type of practice, or professional status. Tip design significantly influences ledge formation. Sharp cutting tips are more likely to cause ledges than modified non-cutting tips. Previous studies reported biconical tips showed the least tendency for ledge formation compared with conical and pyramidal tips due to reduced transitional angles and dual guiding faces (17 , 23). Procedural errors can be further minimized by dulling the flutes on the outer surface of the apical third and the inner surface of the middle third using a diamond file (22). All currently available NiTi rotary instruments possess non-cutting tips. Significant differences in responses were associated with clinical experience, whereas practice type and professional status showed no significant influence. Most participants in this survey believed that files with a negative rake angle are more likely to produce ledges, which contradicts existing literature. Although a positive rake angle enhances cutting efficiency, it may increase the risk of ledge formation. In contrast, files with a negative rake angle exhibit lower cutting efficiency but are less likely to cause ledging (21). Responses to this question were significantly influenced by training, clinical experience, and professional background. Limitations This cross-sectional questionnaire-based study relied on self-reported responses and may therefore be subject to response bias. In addition, the assessment reflected participants' knowledge and reported practices rather than actual clinical performance, which may limit the generalizability of the findings. Further multicenter studies with larger and more diverse samples are recommended.

## Conclusions

Based on the results of this survey, knowledge regarding endodontic mishaps was generally adequate among both specialists and general dental practitioners, including awareness of NiTi instruments and contemporary materials such as MTA. However, understanding of the etiology of apical perforation, routine use of electronic apex locators, and perception of factors contributing to ledge formation were comparatively limited among general practitioners. Attitudes toward the prevention and management of mishaps were favorable in both groups. These findings emphasize the need for targeted continuing dental education programs to address existing gaps, improve clinical competence, and enhance patient outcomes.

## Figures and Tables

**Table 1:** Comparative analysis by years of experience.

Questionnaire	Options	<1 yearsN=28	1-5 yearsN=110	6-10 yearsN=41	>10 yearsN=23	p-value
The most common cause of ledge formation during root canal treatment is?	Use of stiff instruments in curved canals	12	92	38	15	0.001*
Apical perforation most commonly occurs due to	Using rotary files in curved canals without establishing a glide path, Over-preparation beyond the apical foramen, Improper access cavity design . All of the above	19	64	22	14	0.466
Which of the following is a key sign indicating ledge formation?	Sudden loss of working length	18	92	34	18	0.49
Which material is considered the gold standard for perforation repair?	MTA	27	108	41	22	0.35
Which imaging technique is best for diagnosing ledge formation & perforations?	CBCT	14	83	31	17	0.54
Ledge Formation Is Unavoidable Complication in Curved Canals	Disagree	6	46	19	11	0.29
It is better to refer a perforation case to an endodontist rather than attempt repair without experience.	Agree	28	108	41	22	0.55
Do you believe ledge formation can be prevented by use of Ni-Ti instruments?	Agree	24	106	41	21	0.001*
Do you routinely pre-curve stainless steel hand files for curved canals?	Yes	19	95	36	19	0.86
Do you use apex locator in every case?	Sometimes	7	53	24	9	0.049*
Which cleaning and shaping technique is recommended to prevent ledge formation?	Anti-curvature filing, Balanced force technique, Crown-downAll of the Above	16	66	21	18	0.40
What is the primary goal in managing an apical perforation?	Seal Communication with Peri Radicular Tissue	17	78	32	18	0.02*
Which instrument is most likely to cause ledge formation?	K file	12	60	20	14	0.78
Based on tip design of instrument, which produces more ledge?	Pyramidal tip	14	98	41	20	0.02*
Which rake angle of an endodontic instrument is most likely to produce a ledge?	Negative Rake Angle	11	69	33	13	0.31

1

**Table 2:** Comparative analysis by professional status.

Questionnaire	Options	GeneralPractitionerN=105	Endodontist & PGN=97	p-value
The most common cause of ledge formation during root canal treatment is?	Use of stiff instruments in curved canals	89	68	0.030*
Apical perforation most commonly occurs due to	Over-preparationbeyond the apicalforamen	5	21	0.001*
Which of the following is a key sign indicating ledge formation?	Sudden loss of working length	83	79	0.832
Which material is considered the gold standard for perforation repair?	MTA	103	95	0.096
Which imaging technique is best for diagnosing ledge formation & perforations?	CBCT	73	72	0.28
Ledge Formation Is Unavoidable Complication in Curved Canals	Disagree	46	36	0.56
It is better to refer a perforation case to an endodontist rather than attempt repair without experience.	Agree	105	94	0.109
Do you believe ledge formation can be prevented by use of Ni-Ti instruments?	Agree	103	89	0.036*
	Disagree	2	8	
Do you routinely pre-curve stainless steel hand files for curved canals?	Yes	90	79	0.024*
	No	0	5	
Do you use apex locator in every case?	Always	7	29	0.001*
Which cleaning and shaping technique is recommended to prevent ledge formation?	All of the Above	52	69	0.001*
What is the primary goal in managing an apical perforation?	Seal Communication with Peri Radicular Tissue	69	76	0.10
Which instrument is most likely to cause ledge formation?	K file	64	42	0.281
Based on tip design of instrument, which produces more ledge?	Pyramidal tip	94	79	0.92
Which rake angle of an endodontic instrument is most likely to produce a ledge?	Positive Rake Angle	27	40	0.006*

2

**Table 3:** Comparative analysis by type of practice.

Questionnaire	Options	AcademicInstitution(103)	PrivatePractice(99)	p-value
The most common cause of ledge formation during root canal treatment is?	Use of stiff instruments in curved canals	74	83	0.044*
Apical perforation most commonly occurs due to	Using rotary files in curved canals without establishing a glide path, Over-preparation beyond the apical foramen, Improper access cavity design, All of the above	62	57	0.059
Which of the following is a key sign indicating ledge formation?	Sudden loss of working length	85	77	0.557
Which material is considered the gold standard for perforation repair?	MTA	101	97	0.968
Which imaging technique is best for diagnosing ledge formation & perforations?	CBCT	76	69	0.350
Ledge Formation Is Unavoidable Complication in Curved Canals	Disagree	44	38	0.635
It is better to refer a perforation case to an endodontist rather than attempt repair without experience.	Agree	100	99	0.087
Do you believe ledge formation can be prevented by use of Ni-Ti instruments?	Agree	98	94	0.949
Do you routinely pre-curve stainless steel hand files for curved canals?	Sometimes	11	17	0.042*
Do you use apex locator in every case?	Sometimes	42	51	0.072
Which cleaning and shaping technique is recommended to prevent ledge formation?	Anti-curvature filing, Balanced force technique, Crown-down.All the above	59	62	0.342
What is the primary goal in managing an apical perforation?	Seal Communication with Peri Radicular Tissue	75	70	0.525
Which instrument is most likely to cause ledge formation?	K file	50	56	0.444
Based on tip design of instrument, which produces more ledge?	Pyramidal tip	85	88	0.331
Which rake angle of an endodontic instrument is most likely to produce a ledge?	Negative Rake Angle	54	72	0.008*

3

## References

[1] Jafarzadeh H, Abbott PV (2007). Ledge formation: review of a great challenge in endodontics. J Endod.

[2] Alshehri MM, Alhawsawi BF, Alghamdi A, Aldobaikhi SO, Alanazi MH, Alahmad FA (2024). The management of root perforation: a review of the literature. Cureus.

[3] Estrela C, Pécora JD, Estrela CRA, Guedes OA, Silva BS, Soares CJ (2017). Common operative procedural errors and clinical factors associated with root canal treatment. Braz Dent J.

[4] Bhuva B, Ikram O (2020). Complications in endodontics. Prim Dent J.

[5] Abdulrab S, Alaajam W, Al-Sabri F, Doumani M, Maleh K, Alshehri F (2018). Endodontic procedural errors by students in two Saudi dental schools. Eur Endod J.

[6] Chaniotis A, Ordinola-Zapata R (2022). Present status and future directions: management of curved and calcified root canals. Int Endod J.

[7] Barbosa MCRF, Ribeiro EE, Pinto KP, Barbosa AFA, Silva EJNL, Sassone LM (2025). Influence of engine-driven NiTi files on the effectiveness and technical quality of endodontic treatment performed by undergraduate students: a systematic review and meta-analysis. Int Endod J.

[8] Tabassum S, Zafar K, Umer F (2019). Nickel-Titanium Rotary File Systems: What’s New?. Eur Endod J.

[9] Berutti E, Alovisi M, Moccia E, Carossa M, De Caro G, Roccuzzo A (2022). Micro-computed tomographic evaluation of endodontic ledge position in relation to canal curvatures. BMC Oral Health.

[10] Sarao SK, Berlin-Broner Y, Levin L (2020). Occurrence and risk factors of dental root perforations: a systematic review. Int Dent J.

[11] Shokri A, Eskandarloo A, Noruzi-Gangachin M, Khajeh S (2015). Detection of root perforations using conventional and digital intraoral radiography, multidetector computed tomography and cone-beam computed tomography. Restor Dent Endod.

[12] Bilaiya S, Patni PM, Jain P, Pandey SH, Raghuwanshi S, Bagulkar B (2020). Comparative evaluation of accuracy of Ipex, Root ZX Mini, and Epex Pro apex locators in teeth with artificially created root perforations in presence of various intracanal irrigants. Eur Endod J.

[13] Chang YC, Wang TY (2025). Effectiveness of microscope-assisted root canal treatment in permanent posterior teeth: a retrospective cohort study. J Dent.

[14] Shekarbaghani SA, Bolhari B, Khalilak Z (2024). The effect of different root canal irrigations on the accuracy of apex locators: a systematic review. J Clin Exp Dent.

[15] Cassim I, van der Vyver PJ (2013). The importance of glide path preparation in endodontics: a consideration of instruments and literature. S Afr Dent J.

[16] Schäfer E, Dammaschke T (2009). Development and sequelae of canal transportation. Endod Topics.

[17] Lambrianidis T (2006). Ledging and blockage of root canals during canal preparation: causes, recognition, prevention, management, and outcomes. Endod Topics.

[18] Dong X, Xu X (2023). Bioceramics in endodontics: updates and future perspectives. Bioengineering (Basel).

[19] Valverde Haro HP, Rupaya CRG, Alves FRF (2024). Procedural errors detected by cone beam tomography in cases with indication for retreatment: an in vivo cross-sectional study. Restor Dent Endod.

[20] Paiva HC, Akisue E, Ferreira FP, Matos KN, Al Zaibak H, Scardini IL (2023). The use of cone beam computed tomography by Brazilian endodontists: a questionnaire-based survey. J Clin Exp Dent.

[21] Ramachandran T, Kumari VA, Porkodi I (2021). Factors influencing ledge formation and its management in endodontics. Indian J Forensic Med Toxicol.

[22] Ansari I, Maria R (2012). Managing curved canals. Contemp Clin Dent.

[23] Kandaswamy D, Venkateshbabu N, Porkodi I, Gali P (2009). Canal-centering ability: An endodontic challenge. J Conserv Dent.

[24] Pisano M, Sangiovanni G, Frucci E, Scorziello M, De Benedetto G, Iandolo A (2024). Evaluation of the accuracy of electronic apex locators in modern endodontics: an umbrella review. Medicina (Kaunas).

[25] Minu J, Susila AV, Veronica AK (2026). A novel non-invasive radiographic working length determination method: a randomized controlled clinical trial. Endodontology.

[26] Jiménez-Sánchez MC, Segura-Egea JJ, Zarza-Rebollo A, Areal-Quecuty V, Montero-Miralles P, Martín-González J (2021). Use of contemporary technologies and new materials in undergraduate endodontics teaching. J Clin Exp Dent.

[27] Sakkir N, Thaha KA, Nair MG, Joseph S, Christalin R (2014). Management of dilacerated and S-shaped root canals: an endodontist’s challenge. J Clin Diagn Res.

[28] El-Kishawi M, Khalaf K (2021). An update on root canal preparation techniques and how to avoid procedural errors in endodontics. Open Dent J.

[29] Baroudi K, Samir S (2016). Sealing ability of MTA used in perforation repair of permanent teeth: literature review. Open Dent J.

[30] Nagmode P, Janbandhu P, Jagtap A, Basatwar H, Godge S, Shinde S (2023). A scanning electron microscopic study evaluating the sealing ability of MTA, BiodentineTM, and new light-cure MTA used for furcal perforation repair. J Clin Exp Dent.

